# Incidence of cerebral small vessel disease-related MR markers in the Swedish general population ‘Good Aging in Skåne’(GÅS) study

**DOI:** 10.1007/s00415-024-12562-3

**Published:** 2024-07-18

**Authors:** Sölve Elmståhl, Katarina Ellström, Arkadiusz Siennicki-Lantz, Jimmy Lätt, Sven Månsson, Tomas Månsson, Kasim Abul-Kasim

**Affiliations:** 1 Department of Clinical Sciences, Division of Geriatric Medicine, Lund University, Skåne University Hospital, Jan Waldenströmsgata 35, 205 02 Malmö, Sweden; 2 Department of Medical Imaging and Physiology, Skåne University Hospital, Lund, Sweden; 3 Medical Radiation Physics, Department of Translational Medicine, Lund University, Malmö, Sweden; 4 Department of Clinical Sciences, Division of Diagnostic Radiology, Lund University, Malmö, Sweden

**Keywords:** Cerebral small vessel disease, Risk-factor, Incidence, Epidemiology

## Abstract

**Background and objectives:**

Cerebral small vessel disease (CSVD) is associated to cognitive decline and dementia. Neuroimaging changes of CSVD are highly prevalent above 80 years. Only few studies report on incidence of CSVD in high age. We have investigated the incidence and prevalence of magnetic resonance imaging (MRI) markers of CSVD and risk factors in the general older population.

**Methods:**

As part of the general population Good Aging in Skåne cohort study (GÅS), 241 persons (mean age 76.3 years) underwent two brain MRI, 3-T scanner with a mean interval of 5.9 years. The incidence of white matter hyperintensities (WMH), lacunar infarction, cerebral atrophies and cerebral microbleeds (CMB) were calculated and the relationship to risk factors analysed by a multivariate regression analysis. Medial temporal lobe atrophy (MTA) was graded according to Scheltens’18 scale and CMB were defined as having > 1 small (0.2–0.5 cm) hypointense lesion.

**Results:**

The 6-year incidence of CMB, WMH and MTA were, 19%, 17% and 13% respectively, corresponding to 170/1,000 py., 172/1,000 py., and respectively 167/1,000 py. The incidence of CSVD according to the modified STRIVE score was 33%, 169/1,000 py and the prevalence at baseline was 73%. Moderate to high intake of alcohol was related to increased incidence of MTA and higher STRIVE score. Exposure to smoking was related to higher incidence of CMB and higher STRIVE score, adjusted for other known risk factors.

**Conclusion:**

CSVD is highly prevalent in the general older population and the 6-year incidence of WMH, CMB and MTA ranges from 13 to 19 percent. The modifiable lifestyle factors: smoking, and moderate alcohol intake are related to incident CSVD.

## Introduction

Cerebral small vessel disease (CSVD) involves small cerebral vessels that perfuse white matter tracts, basal ganglia, thalamus, and pons. The condition increases with age, affecting about 5% of people aged 50 years, to almost 100% of people older than 90 years [1]. CSVD can be asymptomatic. Clinical manifestations as cognitive decline, dementia, stroke, depressive mood and gait disturbances have been associated to CSVD, although the consequences are unknown and cognitive healthy persons can show these changes why the clinical relevance has been highlighted [2]. The neuroimaging markers of CSVD include small infarctions, white matter hyperintensities (WMH), lacunar infarctions, cerebral microbleeds (CMB), enlarged perivascular spaces, and cerebral atrophy [3, 4]. The interest of CSVD has increased globally since the condition is one of the single most common cause of stroke injury and risk factor for cognitive impairment [5]. The most common pathologies are cerebral amyloid angiopathy and hypertensive related small vessel disease. Vascular risk factors are associated to CSVD such as hypertension, hypercholesterolemia and diabetes, but less have been studied on modifiable lifestyle factors like smoking, alcohol and physical activity [6].. Furthermore, results on alcohol intake are contradictory [6].

Prevalence of CSVD increases with age, especially WMH and CMB. No sex differences are noted (1). WMH progression predicted incident all-cause dementia during 14-year follow-up [7]. A similar study, the Rotterdam Scan study, reported progression of periventricular white matter lesions among 27% and incident CMB among 10% with repeated MRI of older subjects during a 3.4-year follow-up (mean age 71 yrs.) [8, 9]. The most common clinical manifestations are with lacunar stroke and cognitive impairment but in the early stages of CSVD there are no symptoms, only imaging changes. Few studies describe incidence of imaging CSVD including WMH, CMB, lacunar infarction and atrophy. The underlying aetiology of CMB depends on spatial distribution with deep/infratentorial CMB more related to hypertensive vasculopathy. This spatial distribution was not specifically analysed in this study.

We investigated the prevalence and six-year incidence of neuroimaging markers of CSVD in a large population-based sample of older subjects. Furthermore, we studied the relationship between incident lesions and modifiable risk factors.

## Methods

The study is based on the ongoing population-based cohort study “Good Aging in Skåne” (GÅS), part of the “Swedish National study on Aging and Care” (SNAC) [10, 11]., investigating age-related changes on MRI of the brain in an observational cohort design. We have previously reported prevalence of CSVD at the baseline examination [12].. Of the 407 eligible persons from baseline, 241 (59%) participated in a 6-year follow-up during 2022 to 2023. 34 subjects were deceased, 10 subjects were unable to reach, 6 subjects had pacemaker, 5 subjects had dementia and 111 declined to participate. The survey included physical and medical examination by a physician, medical history and information from medical records, anthropometrics, and lifestyle factors from a self-reported questionnaire in an outpatient setting. Cystatin C and creatinine were analysed and estimated glomerular filtration (eGFR) was calculated from the well-established chronic kidney disease epidemiology collaboration (CKD-EPI) formula adjusted for age and sex [13].CKD is defined as having an eGFR < 60 ml/min/1.73 m^2^. Coronary artery disease included heart failure, angina pectoris, atrial fibrillation and presence of cardiac vascular implants or grafts. Stroke/TIA included nontraumatic intracranial haemorrhage, cerebral infarction, and transient ischemic episodes. All diagnoses were derived from the medical examination and medical records and the National Inpatient and Outpatient Register and coded according to the International Classification of Diseases, ICD-9, and ICD-10 to avoid recall bias.

Smoking habits were categorized into three groups, regular/occasional smoking, former smokers, and nonsmokers. Physical activity was divided into two categories, low to medium activity referring to a sedentary lifestyle or light activity for 2–4 h weekly, and high activity with gardening, running or other strenuous activities for > 3 h weekly. Alcohol intake the past year was categorized into three groups, several times a week, less than once a week and teetotaller. Cognitive function was assessed by the Mini Mental State Examination (MMSE) were < 24 score indicate impaired cognition, 25–27 intermediate cognition and scores between 28–30 normal cognition.

MRI examination with a three Tesla MRI (General Electric, discovery MR 750w) included axial T2‐weighted fluid‐attenuated inversion recovery (T2 FLAIR), axial diffusion‐weighted images (DWI), and axial susceptibility‐weighted angiography (SWAN). SWAN sequences were acquired at two settings: 3‐mm‐thick SWAN images and 5‐mm‐thick phase images in order to differentiate blood from calcifications. Furthermore, sagittal T1‐weighted 0.9‐mm isotropic 3D fast spoiled gradient echo (3D‐FSPGR) images were performed and reconstructed in the axial and coronal planes. All MR images were assessed by an experienced neuroradiologist, by KAK (one of the authors) at baseline and by ES at the six-year follow-up, blinded for clinical information. White matter hyperintensities (WMH) changes were assessed by FLAIR sequences. Presence of WMH was graded by the Fazekas scale, where a score of > 2 was considered pathological for WMH [14].. Lacunar infarctions were defined as having > 1 ischemic infarction (< 1.0 cm) in the deep white matter, the basal ganglia and pons [15].. Cerebral microbleeds (CMB) were defined as having > 1 small (0.2–0.5 cm) hypointense lesion [15] using the SWAN sequence. Medial temporal atrophy (MTA) was graded according to Scheltens’18 scale, parietal atrophy graded according to Koedam scale [16]., global cortical atrophy (GCA) assessed according to the Pasquier scale [17]and specific types of atrophy, including frontal cortical, temporal cortical and frontotemporal atrophy were assessed. A modified STRIVE variable from Standards for reporting vascular changes on neuroimaging (STRIVE and STRIVE-2) presented by Wardlaw [3, 18] was defined as presence of at least one of the following: Fazekas scale ≥ 2, ≥ 1 lacunar infarct, ≥ 1 CMB, cortical atrophy, and specific atrophy. The same MRI protocol was used at both examinations.

MRI diagnoses of CMB have proven to be reproducible with good to very good intra‐ and inter-rater agreement (intra-rater kappa = 0.85 [95% confidence interval (CI) 0.77‐0.93]; interrater kappa = 0.68 [95% CI: 0.58‐0.78]) in previous studies [19]..

Incidence and prevalence of CSVD were calculated and a logistic multivariate regression model for analyses of association between incident cases of CSVD and established risk factors such as age, lifestyle factors, coronary and cerebrovascular diseases, diabetes, hyperlipidaemia, and hypertension. Chi square statistics were used for analyses of differences between participants and non-participants at follow-up. All statistical analyses were performed using the software IBM SPSS version 27. The statistical significance level was set at 5%. The STROBE checklist for cohort studies was used.

## Results

The mean age of the study sample at baseline was 76.3 years and mean follow-up time was 5.9 years. A higher proportion of the participants at follow-up had high physical activity, were non-smokers and reported moderate to high alcohol intake compared to non-participants, see Table 1.

**Table 1:** Baseline characteristics at the first examination of the study population from the Good Aging in Skåne study (GÅS), part of the Swedish National study on Aging and Care

	Study population with second MRI (n = 241)	Non-participants at follow-up (n = 167)	*p* value
Age (mean/SD	76.3 /3.29	77.9/4.02	ns
Education (%)			ns
Elementary school	33	36	
Secondary school	35	38	
University > 1 yr	32	26	
Smoking (%)			ns
Never smoker	42	34	
Former smoker	49	52	
Current smoker	9	14	
Alcohol intake (%)			< 0.001
Teetotaller	9	15	
Less than once a week	43	58	
Several times a week	48	27	
Physical activity past year (%)			0.01
Mostly sedentary	6	14	
Light activity	50	55	
Strenuous activity	44	32	
BMI (%)			ns
25–29 overweight	47	49	
> 30 obese	19	19	
Diabetes, type 1 or 2 (%)	15	22	ns
Hypertension (%)	56	67	0.02
Hyperlipidaemia (%)	42	50	ns
Coronary artery disease (%)	35	41	ns
Stroke/TIA	12	13	ns
Cognition, MMSE (%)			ns
28–30	71	63	
25–27	24	28	
< 24	4	9	
SBP/DBP mm Hg mean/SD	134/75 15.1/8.5	137/70 16.7/9.8	ns/ns
eGFR^1^ mean/SD (ml/min/1.73m^2^)	69.6/12.4	66.4/14.4	ns
eGFR < 60 ml/min/1.73m^2^	20%	28%	ns

^1^eGFR estimated glomerular filtration calculated from creatinine and cystatin C using the CKD-EPI formula

*SBP* systolic blood pressure, *DBP* diastolic blood pressure, *BMI* body mass index, *MMSE* Mini Mental State Examination

Prevalence of the MRI findings at the baseline survey and their 6-year incidence are presented in Table 2. CMB, WMH and MTA had the highest 6-year incidence, 19%, 17% and 13% respectively, corresponding to 170/1000 py., 172/1000 py., and respectively 167/1000 py, see Table 2. The incidence of CSVD according to the modified STRIVE was 33%, 169/1000 py., and the prevalence of STRIVE was 73% at baseline. Higher numerically incidence was noted for the following CSVD lesions: WMH, CMB, GCA, MTA, lacunar infarction and specific atrophy, among older participants at age 76–87 yr., compared to the 72–75 yr. group, with a mean difference of incidence ranging from 8/1000 py. to 17/1000 py., as presented in Table 2.

**Table 2:** Prevalence and 6-year incidence of cerebral small vessel disease (CSVD) in relation to age from repeated MRI. Data from the Good Aging in Skåne study (GÅS) part of the Swedish National study on Aging and Care

	Incident cases during 6-year follow up					
	Incident cases (%)	Number of persons	Mean p.y	Total p.y	6-year incidence /1000 p.y	Prevalence at baseline (%)
Total study sample	100%	241	5.88	1.418	–	
Men (%)/women (%)		99 (41)/142 (59)	5.92/5.86	566/832	–	
72–75 yr. (%)		56 (43)/73 (57)	7.02/7.01	393/512	–	
76–87 yr. (%)		43 (38)/69 (62)	7.01/7.01	301/484		
*MRI findings*						
WMHs (%)^1^ (all)	17%	40	5.81	232	172	81%
72–75 yr		20	6.02	120	167	78%
76–87 yr		20	5.61	112	179	84%
Lacunar infarction^2^	9%	20	6.00	120	167	11%
72–75 yr		9	6.31	57	158	
76–87 yr		11	5.76	63	175	
CMBs^3^	19%	27	5.88	159	170	28%
72–75 yr		16	6.07	97	165	
76–87 yr		11	5.66	62	177	
Global cortical atrophy^4^	3%	8	5.59	45	178	11%
72–75 yr		4	5.76	23	174	
76–87 yr		4	5.31	22	182	
MTA^5^	13%	32	5.99	192	167	70%
72–75 yr		21	6.17	130	162	
76–87 yr		11	5.65	62	177	
Specific atrophy^6^	4%	7	5.87	41	171	27%
72–75 yr		4	6.04	24	167	
76–87 yr		3	5.68	17	176	
Parietal atrophy	5%	12	5.75	69	174	32%
72–75 yr		7	5.57	39	179	
76–87 yr		5	6.01	30	167	
Precuneus atrophy	4%	9	6.20	56	161	30%
72–75 yr		6	6.14	37	162	
76–87 yr		3	6.32	19	158	
WMC pontine	9%	18	6.00	108	167	14%
72–75 yr		9	5.76	52	173	
76–87 yr		9	6.26	56	161	
Modified STRIVE^7^	33%	20	5.89	118	169	73%
72–75 yr		16	6.11	98	163	
76–87 yr		4	5.67	23	174	

^1^WMHs white matter hyperintensities defined as Fazekas scale ≥ 2

^2^Lacunar infarcts defined as presence of ≥ 1 ischemic infarction (< 1.0 cm)

^3^CMBs cerebral microbleeds defined as defined as presence of ≥ 1 small hypointense lesion (0.2–0.5 cm)

^4^Global cortical atrophy (GCA) ≥ 1 according to Pasquier scale

^5^MTA medial temporal lobe atrophy

^6^Specific atrophy defined as at least one of the following entities global cortical atrophy (GCA) ≥ 1 according to Pasquier scale, Koedam parietal score ≥ 1 frontal/frontotemporal/temporal atrophy and ≥ 1

^7^Presence of CSVD according to the modified STRIVE variable was defined as the presence of at least one of the following entities Fazekas scale ≥ 2, ≥ 1 lacunar infarct, ≥ 1 CMB, cortical atrophy and specific atrophy

Sixteen out of the 241 subjects (7%) had regression of one or more of the CSVD lesions: reduction in number of lacunar infarctions in 11 subjects, precuneus atrophy in 3 subjects, Koedam parietal atrophy, specific atrophy, and number of CMB in 2 subjects each, and GCA, WMH and MTA in 1 subject.

Table 3 presents the relationship and odds ratios between life-style factors and the incidence of CSVD lesions 6 years later. Moderate to high intake of alcohol was related to increased incidence of MTA and higher STRIVE score, while exposure to smoking was related to a higher incidence of CMB and higher STRIVE score, adjusted for other known risk factors.

**Table 3:** Odds ratios (OR) of incident cerebral small vessel disease (CSVD) during 6-year follow-up given exposure to smoking and alcohol from a multivariate regression analysis, adjusted for age, hypertension, diabetes, cardiovascular disease, stroke/TIA, chronic kidney disease (eGFR < 60 ml/min/1.73 m^2^) and physical activity

Incident MRI features of CSVD	OR	S.E	*p* value
CMB^1^			
Smoking^5^	2.73	0.44	0.021
MTA^2^			
Alcohol intake^4^	2.58	0.43	0.029
Modified STRIVE^3^			
Smoking	3.88	0.55	0.013
Alcohol intake	3.39	0.57	0.032
Stroke/TIA	1.03	0.64	0.106

^1^CMBs cerebral microbleeds defined as defined as presence of ≥ 1 small hypointense lesion (0.2–0.5 cm)

^2^MTA medial temporal lobe atrophy

^3^Presence of CSVD according to the modified STRIVE variable was defined as the presence of at least one of the following entities Fazekas scale ≥ 2, lacunar infarct ≥ 1, CMB, cortical atrophy and specific atrophy ≥ 1

^4^Alcohol several times a week *vs* less than one a week/teetotaller

^5^Smoking former/current *vs* never smoker

## Discussion

Incidence of CSVD is common and a third of an older general population in this study develop CSVD during a 6-year follow-up according to a modified STRIVE score and 13–19% develop either CMB, MTA or WMH. The incidence of CSVD is related to smoking and moderate to high alcohol intake.

Most previous studies on CSVD are cross-sectional, include somewhat younger populations up to 70 years, and presented variation in incidence and prevalence which could depend on difference in MRI techniques used and definitions of imaging findings. The Rotterdam Scan study reported an overall 16% incidence of CMB in 70–79-year-old participants, which is like our findings [9, 20]. Also, the prevalence of CMB at baseline examinations was similar between these two studies: 32% compared to 28% in our study. The incidence of lacunar infarction in the Three city-Dijon study with 4-year follow-up was 10%, which is similar to 9% in this study [21]. Previous studies have highlighted the need for age stratification instead of incorporating age as an independent variable since CSVD is not consistent among age groups [22].The burden of CSVD increases with age and the annual incidence noted in this study is about 2% in the general older population and similar or higher than many of other prevalent chronic disorders among older subjects. For example, the annual incidence of hip fracture is 1.% in women and 0.7% in men above 70 years [23] and the incidence of stroke between 0.7 and 1.0% for those aged 65–74 in US [24]. This study report that the high incidence of CSVD remains above 80 year of age.

It is important to identify possible modifiable risk factors of CSVD to prevent the condition. A recent meta-analysis identified hypertension, diabetes, smoking and hyperlipidaemia as risk factors for CSVD (6). Alcohol consumption is a significant risk factor for stroke [25], but results on association to CSVD are contradictory. High intake of alcohol is related to cerebral atrophy and involvement of the insular cortex, part of the temporal lobe and low to moderate intake of alcohol is associated with a reduced risk of ischemic stroke [26]. We noted an increased risk for moderate alcohol consumption in relation to MTA. The effect of smoking was studied previously in pooled analyses indicating an effect on both WMH and lacunar infarction and in accordance with the findings in the present study [6].

The findings in this study indicate that the longstanding effect of exposure to the modifiable lifestyle factors smoking and alcohol remains at high age and could be detected as increased incidence of CSVD above 75 years of age.

We have previously reported on the cross-sectional findings of CSVD and at follow-up in this study the participants had higher alcohol intake and higher physical activity but less hypertension than the non-participants [12].A selection bias with more healthier subjects cannot be ruled out and the observed incidence might underestimate true incidence of CSVD. Furthermore, the high age of the study population ranging from 70 to 87 years at inclusion also cause additional attrition bias of the detrimental effects of risk-factors like high exposure to smoking and alcohol since those subjects to a higher degree are deceased. The improved treatment the past decades of several cardiovascular risk factors like hypertension and diabetes, RAS inhibitors that reduce progression of chronic kidney disease and cardiovascular events and cessation of smoking will most likely slow down the progression of CSVD in the very old. Future studies will evaluate these effects on newer birth cohorts with changed life-time exposure of risk-factors on CSVD.

The findings from the MMSE describing the general cognitive level among the participants indicates that the vast majority had a high functioning level (> 27 MMSE score) despite the high prevalence of CSVD related MRI markers. The relationship between imaging findings and change in cognitive function, especially executive functions also need to be further explored. There are few treatments for established CSVD, and further studies are needed to understand the pathogenesis and factors influencing progression [27]. The absence of autopsy is a limitation since it could have reduced possible misinterpretations of the MR related CVSD lesions and future studies would benefit with information on ApoE status and family history of cognitive decline or PD. Lack of analyses of superficial siderosis as a MR marker of cerebral amyloid angiopathy is another limitation.

In conclusion, CSVD is highly prevalent in the general older population above 80 year of age and the 6-year incidence of WMH, CMB and MTA ranges from 13–19%. The modifiable life-style factors smoking, and moderate alcohol intake are related to incident CSVD.
